# Retraction Note for Singh *et al.* Performance evaluation of DNA Motif discovery programs Published on December 31, 2018

**DOI:** 10.6026/97320630014594

**Published:** 2018-12-31

This adds a note to the retraction notice “ Performance evaluation of DNA Motif discovery programs” in volume 11 and Issue 11 on page 516

## Note to retarction notice

The article 1 retracted 2 by the journal (Bioinformation) was communicated by the first author (Chandra Prakash Singh) without our knowledge, willingness and consent. Hence, we the following report our innocence in this context.

Feroz Khan, Department of Metabolic and Structural Biology, CSIR–Central Institute of Medicinal and Aromatic Plants, Lucknow-226015 (U.P.) India

Bhartendu Nath Mishra, Department of Biotechnology, Institute of Engineering and Technology, Dr. A.P.J. Abdul Kalam Technical University, Sitapur Road, Lucknow-226021 (U.P.) India; BN Mishra - E-mail: profbmishra@gmail.com
